# Handedness and midsagittal corpus callosum morphology: a meta-analytic evaluation

**DOI:** 10.1007/s00429-021-02431-4

**Published:** 2021-12-01

**Authors:** René Westerhausen, Marietta Papadatou-Pastou

**Affiliations:** 1 Department of Psychology, University of Oslo, POB 1094 Blindern, 0317 Oslo, Norway; 2 Biomedical Research Foundation of the Academy of Athens, Athens, Greece; 3 School of Education, National and Kapodistrian University of Athens, Athens, Greece

**Keywords:** Handedness, Corpus callosum, Brain asymmetry, Hand preference

## Abstract

Following a series of seminal studies in the 1980s, left or mixed hand preference is widely thought to be associated with a larger corpus callosum than right handedness, influencing the interpretation of findings and various theories related to interhemispheric processing, brain lateralisation, and hand preference. Recent reviews, however, find inconsistencies in the literature and cast doubt on the existence of such an association. The present study was conducted to clarify the relationship between hand preference and callosal morphology in a series of meta-analyses. For this purpose, articles were identified via a search in PubMed and Web Of Science databases. Studies reporting findings relating to handedness (assessed as hand preference) and corpus-callosum morphology in healthy participants were considered eligible. On the basis of a total of *k* = 24 identified studies and databases, random-effects meta-analyses were conducted considering four different group comparisons: (a) dominantly right- (dRH) and left-hand preference (dLH), (b) consistent right (cRH) and non-cRH preference, (c) cRH with mixed-hand preference (MH), and (d) cRH with consistent left-hand hand preference (cLH). For none of these meta-analyses did we find a significant effect of hand preference, and narrow confidence intervals suggest that the existence of population effects larger than 1% explained variance could be excluded. For example, considering the comparison of dRH and dLH (*k* = 14 studies; 1910 dRH and 646 dLH participants) the mean effect size was Hedge’s *g* = 0.016 (95% confidence interval: − 0.12 to 0.15; explained variance: < 0.001%). Thus, the common practice of assuming an increase in callosal connectivity based on mixed or left hand preference is likely invalid.

**Supplementary Information:**

The online version contains supplementary material available at 10.1007/s00429-021-02431-4.

## Introduction

The corpus callosum, formed by a bundle of axons, is essential for the coordination and integration of information processing between and across the two cerebral hemispheres (Gazzaniga 2000). Consequently, it has long been suggested that inter-individual differences in callosal morphology are related to differences in functional hemispheric asymmetries (Galaburda et al. 1990; Ringo et al. 1994; Witelson and Nowakowski 1991). Recent neuroimaging studies in general support this notion as they have shown an association between measures of corpus callosum connectivity and the distribution of neuronal processing between the hemispheres (e.g., Haberling et al. 2011; Josse et al. 2008; Karolis et al. 2019; Labache et al. 2020; Westerhausen et al. 2006).

Hand preference, arguably the most salient functional asymmetry with almost 9 out of 10 individuals being right-handed (Papadatou-Pastou et al. 2020), has also been frequently related to differences in corpus callosum structure and function (for review see, Beaton 1997 and Budisavljevic et al. 2020). These studies can be roughly classified into either of two categories: studies that conceptualise handedness based on the hand preferred for most tasks (direction of hand preference, i.e., left vs. right) or the consistency of hand preference across tasks (consistent vs. inconsistent preference). Although both approaches have been frequently employed in the literature (see Supplementary Tables S1 to S4 for an overview), using the consistency of hand preference has been arguably the most influential and it originates in a series of seminal publications by Witelson (1985, 1989) and Witelson and Goldsmith (1991). Witelson had measured the area of the midsagittal corpus callosum in the brain specimen of deceased cancer patients of which handedness had been assessed ante mortem using a handedness questionnaire (Annett 1970). Classifying her patients into consistent right-handers (cRH) and inconsistent mixed handers (MH), she found the total corpus callosum to be larger in the MH group. Witelson interpreted these findings to indicate that a less-lateralized hemispheric organization for hand preference was associated with a stronger or more efficient callosal connectivity than a strongly lateralized organization. Based on this and additional evidence, Witelson derived a theory about the ontogenetic development of functional hemispheric differences (Witelson 1985; Witelson and Nowakowski 1991). That is, she postulated that the degree of functional hemispheric asymmetry (including hand preference) is determined by the magnitude of the loss of callosal axon during late fetal and early postnatal development (LaMantia and Rakic 1990; Innocenti and Price 2005). Strong perinatal axon loss and the resulting limited hemispheric connectivity were thought to promote a strongly lateralised functional brain. A weak axon loss, leaving a comparatively “strong” hemispheric connectivity, in turn, would promote a less pronounced lateralization and hand preference.

Witelson’s theory offers a direct neurophysiological explanation for the association of consistency in hand preference (or hemispheric specialization) and corpus callosum morphology. Consequently, studies frequently refer to Witelson’s findings when explaining behavioral or cognitive differences between right- and non-right handers that might be linked to the corpus callosum, even without measuring the corpus callosum itself (for some recent examples, see e.g., Jasper et al. 2021, Parker et al. 2017, Roberts et al. 2020, Sala et al. 2017, and Zapała et al. 2020). For example, callosal size differences have been used to explain superior episodic-memory performance in mixed- as compared to consistent (right)-handers (Prichard et al. 2013). However, Witelson’s original findings are not necessarily supported by more recent studies and narrative reviews typically summarize the literature as being inconsistent, questioning the existence of a general association (Beaton 1997; Budisavljevic et al. 2020). A quantitative integration of the available studies is, to date, missing.

The aim of the present study was to revisit the question of an association of handedness and corpus callosum morphology using meta-analytic methods. For this purpose, we identified all publications comparing midsagittal corpus callosum morphology in neurologically-healthy participants based on their hand preference. That is, studies were included if they assessed hand preference by self-report (i.e., questionnaire) and used measures of total or subsectional corpus callosum morphology (as volume, area, or thickness) as a dependent variable. As the studies varied in their definition of handedness groups, we could not integrate all data into a single meta-analytic comparison. Rather, we conducted meta-analyses for four different group comparisons: studies that compared participants of (a) dominantly right- (dRH) and dominantly left-hand preference (dLH), (b) consistent right (cRH) with non-cRH preference (NcRH), (c) cRH with mixed-hand preference (MH), and (d) cRH with consistent left-hand preference (cLH). Thus, while meta-analyses (a) and (d) focus on the effect that the direction of hand preference has on the corpus callosum, meta-analyses (b) and (c) also consider the consistency of hand preference. Furthermore, as the interaction of sex and hand preference has received substantial attention in the literature comparing cRH and NcRH samples when explaining total and isthmal corpus-callosum size (Clarke and Zaidel 1994; Habib et al. 1991; Witelson 1989), we conducted a moderator analysis of sex for these comparisons in addition to the overall meta-analysis.

## Material and methods

### Study identification

The study selection was based on a literature search that was conducted on 01. February 2021 on PubMed and Web of Science (Core Collection). The search in PubMed was performed with the search query ("corpus callosum"[All Fields]) AND ("handedness"[All Fields] OR "hand preference" [All Fields]). The search in Web of Science used the search terms (ALL FIELDS: (Corpus callosum) AND ALL FIELDS: (handedness OR hand preference)). Additional studies were identified from the reference list of the selected empirical articles and previous review articles (Beaton 1997; Budisavljevic et al. 2020; Driesen and Raz 1995) and by contacting authors who recently had published corpus callosum data on handedness.

### Record screening, article eligibility check, and inclusion

After removing duplicates, the potential relevance of the study was step-wise evaluated as described in the following. Firstly, title and abstracts were screened to check whether morphological corpus-callosum measures were utilized and that the sample consisted of human participants. If this was the case, the full-text articles were inspected to verify that corpus callosum measures were reported for non-right-handed individuals as well. This step led to the exclusion of studies which had used handedness as an exclusion criterion (typically, excluding non-right handers) or to match participants (without reporting the means for handedness groups). In the case of clinical studies, only the data of healthy control groups were considered relevant. In a final evaluation round, it was determined whether sufficient information was available to calculate an effect size measure and include the data in the quantitative meta-analysis. If this was not the case, the corresponding authors were contacted to obtain the relevant data where possible.

The following list provides an overview of all criteria for screening and eligibility assessment:

- Study languages: publications in English, German, and French were considered.
- Species: only data from human (homo sapiens) samples was included.
- Health: only data from neurologically-healthy individuals were considered. Of note, Witelson’s seminal work examined patients suffering from peripheral (i.e., not directly affecting the central nervous system) cancer (Witelson 1985, 1989; Witelson and Goldsmith 1991) and was included in the analysis.
- Hand preference had to be assessed using self-report measures, i.e. questionnaires or self-identification. Studies using performance or skill measures for assessment were not included due to sparsity.
- Corpus callosum had to be assessed morphologically regarding its midsagittal shape. Studies reporting raw thickness, area, and volume measures were included.
- Reporting of useable arithmetic data (means and standard deviation or standard error) per group or test statistics (e.g., a *t*-value) for the group comparison.

**Fig. 1:**
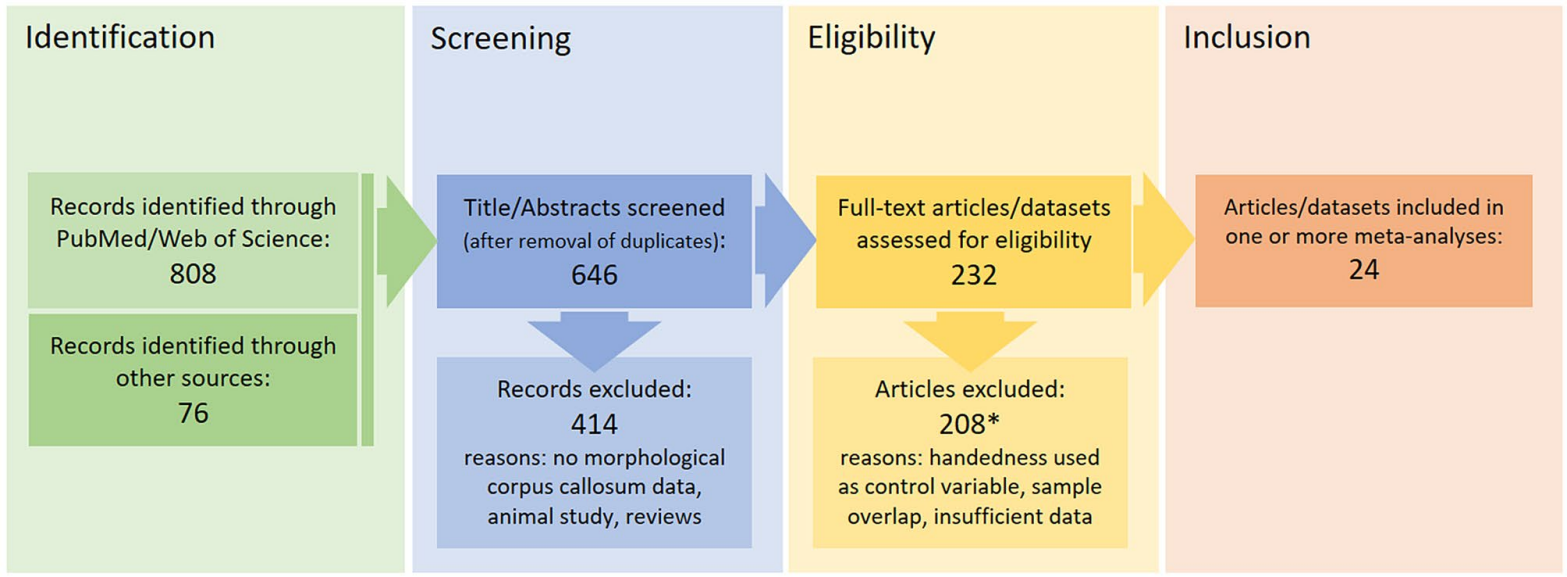
Overview of study identification, screening, and eligibility assessment. All included studies and datasets are presented in Table 1. All studies that were initially considered eligible but did not provide sufficient information for a statistical inclusion or were redundant (i.e., sample overlap) to other included reports can be found in Supplement Table S5 (with a reason for the exclusion)

An overview of the selection procedure and the number of studies excluded on each step can be found in flow-chart (Fig. 1).

### Data extraction

To assure the independence of observation, the overlap in samples across studies was evaluated and it was made sure that each sample was included only once per meta-analysis. In case of such overlap, only most complete data (e.g., the largest sample) was included. For example, the data reported by Cowell and Gurd (2018) represents a sample extension of the data published earlier (Gurd et al. 2013) thus only data from the larger 2018 sample was included. A second example, Witelson and Goldsmith (1991), reports the results only of an extension of the male sample compared to the Witelson (1989) article, therefore the male data was taken from the later and the female data from the earlier publication.

Regarding hand preference, the method of assessment and potential thresholds for group formation were extracted together with the number of individuals in each group. Of note, as is typical in the handedness literature (Ocklenburg and Güntürkün 2018), the studies included used various criteria to define groups of hand preference. Four main approaches were common in the available literature and accordingly considered in the present set of meta-analyses.

- A classification based on the “handedness direction” so that the overall preference across the activities assessed by a handedness questionnaire determines the classification. Typically, a laterality quotient (LQ) of 0 (equivalent to no hand preference, see e.g., Oldfield (1971)) was used to split the sample into a group of dRH and dLH individuals.
- Consistency of hand preference across tasks was considered, by comparing cRH individuals with all other (referred to as NcRH). For this purpose, consistency was defined by the primary studies either by arbitrarily setting a high LQ (e.g., LQ > 80% in Habib et al. (1991)) as cut-off or by qualitative analyses of the response pattern in the handedness questionnaire. The latter approach follows the suggestion by Witelson (1989), who classified participants as cRH if the answers in Annett’s questionnaire (Annett 1970) were “all 'right', or 'right' with some 'either' preferences”. All other individuals (i.e., LQ < 80%; or indicating any ‘left’ preferences) were consequently defined as not being consistent (NcRH).
- Witelson originally picked this solution, as she was not able to find a sufficiently large sample of consistent left-handers to form their own group (Witelson 1985). However, later researchers did so, resulting in the possibility of comparing a cRH group with a group of MH and cLH individuals, as third and fourth comparison, respectively. That is, the NcRH group as defined above, was split into two groups, whereby typically a high negative LQ threshold (e.g., LQ < − 80%) was utilized to separate cLH from the MH group.

**Fig. 2:**
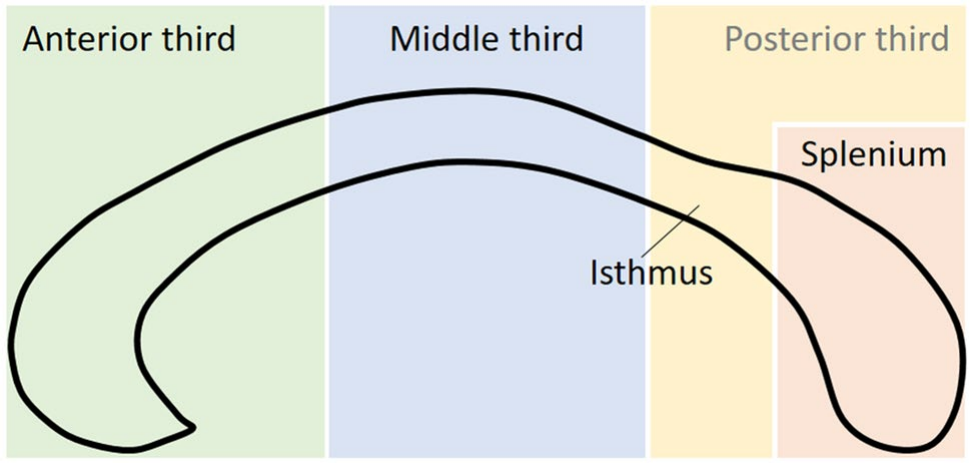
Illustration of corpus callosum subdivision used in the meta-analyses. The approach followed the straight-line method introduced by Jancke et al. (1997). The outline of the corpus callosum (black line) is divided into thirds relative to its anterior–posterior extend. The posterior third is additionally split into a posterior fifth (i.e., the splenium) and the isthmus

Considering the corpus callosum, raw mean and standard deviation of measures of midsagittal callosal morphology (volume, area, thickness) were extracted from the primary articles. The focus on raw measures was necessary as a correction for brain size measures was rarely reported, preventing us from conducting a separate analysis considering brain-size differences. For each study, the method of obtaining the data (i.e., post mortem study or in-vivo MRI; including field strength), the method of callosal segmentation, and the measurement type of the dependent variable (i.e., thickness, area, or volume) was recorded. Where available also measures of corpus callosum subsections were extracted. As the method of dividing the corpus callosum into subsections varies between studies, an attempt was made to apply a common frame of reference across studies. That is, we used the geometrical subdivision schema suggested by Jäncke et al. (1997), which divides the midsagittal corpus callosum surface into four subsections based on the anterior–posterior extend of the structure (see Fig. 2). This schema was considered optimal, as it is sufficiently broad to subsume some of the more fine-grained alternative subdivision schemas, but also includes an isthmus section, which has received particular attention by the literature following Witelson’s original findings (see e.g., Denenberg et al. (1991)). A transfer was made by applying the Jäncke et al. (1997) subdivision scheme on the visual representation of the callosal subdivision provided in the literature and determine correspondence between subdivisions. An illustration of the procedure and the transfer heuristics used can be found in Supplemental material, Fig. S1.

The required corpus callosum data for the meta-analyses was not always readily available in the articles, whereby the following cases were encountered. First, the article did report mean and dispersion of the callosal measures by subgroups (e.g., for female right-handers and male right-handers) but not the entire handedness group. In this case, the data were pooled across the subsamples considering the subsample sizes as weights. Studies where this was the case are indicated in Supplement Tables S1–S4. Second, the article did not report the relevant data, but the author contact was successful. Here the relevant data was either calculated based on the received raw data or the authors were so kind to provide the relevant means and standard deviations. For these cases, a detailed information of the data obtained, the conducted calculations, and results are provided as Supplementary Material Sect. 3. Third, studies did not provide relevant data and authors were non-responsive or no longer traceable. Here, the study was not included in the meta-analyses. A complete list of these studies can be found in Supplement Table S6.

During data extraction, a within study risk-of-bias assessment was done by focussing on the criteria of selective reporting of results. Specifically, it was checked whether the potentially available corpus-callosum data was also reported (see Supplement Table S5). For example, several studies reported to have assessed multiple corpus-callosum subsections but only reported the findings for the significant comparisons (e.g., Hines et al. (1992) and Moffat et al. (1998)). If this was the case, the information was noted and considered when evaluating the outcome of the meta-analyses.

Finally, the sex distribution and age range of each study’s sample were recorded in addition to the variables describing the assessment of handedness and corpus callosum morphology. An overview of these variables can be found in Table 1 as well as in Supplement Tables S1–S4.

**Table 1:** Overview of study inclusion (●) in the meta-analyses considering the total corpus callosum (tcc) or callosal subsections (ss)

#	Study	Assessment methods					Included in meta-analysis								Sample overlap
		HPref^a^	Corpus callosum^b^				dRH vs dLH		cRH vs. NcRH		cRH vs. cLH		cRH vs MH		
		Device	Method (FS)		DV	SD	tcc	ss	tcc	ss	tcc	ss	tcc	ss	
1	Clarke and Zaidel (1994)	EHI	MRI (0.3 T)		A	SL			●	●					Same sample as Clarke et al. (1993)
2	Cowell and Gurd (2018)	EHI	MRI (1.5 T)		A,T	PCA			●	●					Extended sample of Gurd et al. (2013)
3	Denenberg et al. (1991)	BQ	MRI (0.15 T)		A,T	PCA	●	●							Same sample as Kertesz et al. (1987) and Cowell et al. (1993)
4	Haberling et al. (2012)	BQ	MRI (1.5 T)		A	n.a	●		●		●		●		
5	Haberling et al. (2011)	AQ	MRI (1.5 T)		A	n.a	●		●		●		●		
6	HCP, release 2017	EHI	MRI (3 T)		V	SL	●	●	●	●	●	●	●	●	Human Connectome Project Reference Van Essen et al. (2013)
7	Hines et al. (1992)	EHI/C	MRI (1.5 T)		A	CL				●					
8	Jancke et al. (1997)	AQ	MRI (1.5 T)		A	SL			●	●	●	●	●	●	
9	Kertesz et al. (1987)	BQ	MRI (0.15 T)		A	n.a					●		●		Same sample as Denenberg et al. (1991) and Cowell et al. (1993)
10	Labache et al. (2020)	EHI	MRI (3.0 T)		V	n.a	●		●		●		●		
11	Luders et al. (2003)	EHI	MRI (1.5 T)		A	SL					●	●			
12	Martens et al. (2013)	EHI	MRI (1.5 T)		A	SL	●	●							
13	McDowell et al. (2016)	BQ	MRI (1.5 T)		V	SL	●	●	●	●	●	●	●	●	Same sample as Welcome et al. (2009)
14	Moffat et al. (1998)	WQ	MRI (0.5 T)		A	SL	●	●							
15	Morton and Rafto (2006)	SID	MRI (1.5 T)		A	n.a	●								
16	Nasrallah et al. (1986)	AQ	MRI (0.5 T)		A	n.a	●								
17	Ozdikici (2020)	Other	MRI (n.a.)		A	n.a	●								
18	Steinmetz et al. (1992)	AQ	MRI (1.5 T)		A	SL			●	●	●	●	●	●	
19	Tuncer et al. (2005)	BQ	MRI (1.0 T)		A	n.a	●								
20	Van der Haegen et al. (2011)	EHI	MRI (3.0 T)		A	SL	●	●	●	●	●	●	●	●	
21	Westerhausen et al. (2004)	EHI	MRI (1.5 T)		A	SL	●	●	●	●	●	●	●	●	
22	Witelson (1985)	AQ	PM	–	A	SL							●	●	
23/24	Witelson (1989) and Witelson and Goldsmith (1991)	AQ	PM	–	A	SL			●	●					Extended sample of data in Witelson (1985)

^a^Handedness assessment was done with the Annett Inventory (AQ), the Bryden questionnaire (BQ), the Corvitz questionnaire (C), the Edinburgh Inventory (EHI), or the Waterloo questionnaire (WQ)

^b^The corpus callosum was assessed using post mortem (PM) examination or using MRI. In the case of MRI the number in brackets indicates the field strength (FS) of the MRI scanner in Tesla. The dependent variable (DV), i.e. corpus-callosum size, was measured as thickness (T), area (A), or volume (V). In case the corpus callosum was divided into subsection, different routines for subdivision were used: SL = straight-line, PCA = principle-component analysis of thickness measures, RL = the radial method, or CL = curved-line method. The abbreviation “n.a.”, indicates that the respective information was not available

### Statistical analysis

The above-outlined study differences in the handedness classification could not allow for an overall analysis without losing relevant information. We, therefore, decided to conduct a set of four meta-analyses for the following group comparisons: (a) dRH vs. dLH, (b) cRH vs. NcRH, (c) cRH vs. cLH, and (d) cRH vs. MH. Considering (b) we additionally conducted a moderator analysis to explore a suggested interaction of sex and hand preference. The effect size measure Hedges’ *g*, reflecting the standardised mean difference of corpus callosum size, was calculated for each study. In all analyses, the pooled effect size was determined assuming a random-effects model. A random-effects model was preferred, as a fixed-effect model was considered inappropriate given the variability in callosal and handedness measures between studies. The between-study variance (*τ*^2^) was estimated using the DerSimonian-Laird approach and the Hartung-Knapp adjustment was chosen to account for the small number of studies included.

As the number of studies analysing callosal subsections was smaller than the number of studies included in the analysis of the total corpus callosum, we used power analysis to determine the minimal number of studies (*k*_min_) required to provide a power of at least 0.80 for a population effect size of *d* = 0.2 (i.e., a small effect according to Cohen (1992)). For this purpose, we used the average sample size per study and the heterogeneity estimates from the total corpus callosum meta-analyses as best guess values for the subsection analyses. The number of required studies was then determined by iteratively applying the *power.analysis* script provided with the “dmetar” R package (version 0.0.9; (Harrer et al. 2019) with the above parameters. From this, it was determined that the minimum number of studies for comparison (a) was *k*_min_ = 8, for (b) *k*_min_ = 6, for (c) *k*_min_ = 12, and for (d) *k*_min_ = 7. A meta-analytic integration was only performed when the available number of studies was at least equal to the determined *k*_min_.

Visual examination and Egger’s regression were used to inspect the funnel plots of each meta-analysis for a potential small study bias. In addition, if one study had a weight in the analysis of 25% or above, the meta-analysis was repeated without this study to evaluate the stability of the population-effect size, as a form of sensitivity analysis.

All effect size calculations were done using the “esc” R package (version 0.5.1; (Lüdecke 2018). Meta-analytic procedures were conducted using functions of the libraries “metafor” (version 2.4; Viechtbauer 2020), “meta” (version 4.16–1; Schwarzer 2020), and “dmetar” (version 0.0.9; Harrer et al. 2019) using the R environment (version 4.0.3). The reporting of the meta-analysis followed the PRISMA checklist (Page et al. 2021) but was not pre-registered.

## Results

### Descriptive statistics of included studies

Data from *k* = 24 reports and datasets published between 1985 and 2020 were included in one or several meta-analyses as indicated in Table 1. In 9 (38%), 7 (29%), and 5 (21%) of these studies, hand preference was assessed with versions of the Edinburgh Handedness Inventory (Oldfield 1971), the Annett questionnaire (Annett 1970), or the handedness questionnaires suggested by Bryden (1977), respectively. A total of 23 (96%) studies provided measures of total corpus callosum size and 16 (67%) studies provided measures for one or more callosal subsections (of note, one study only provided subsection data; see Table 1 for details). The corpus callosum was assessed by post-mortem morphometry in 3 (12.5%) of these reports and using in-vivo magnetic-resonance imaging in 21 (87.5%) studies. Callosal size was measured as midsagittal area in 21 (87.5%) studies and as volume in 3 (12.5%) studies. Thickness measures were additionally reported in 2 (8%) of the included studies.

### Meta-analysis set for the dRH vs. dLH comparison

**Fig. 3:**
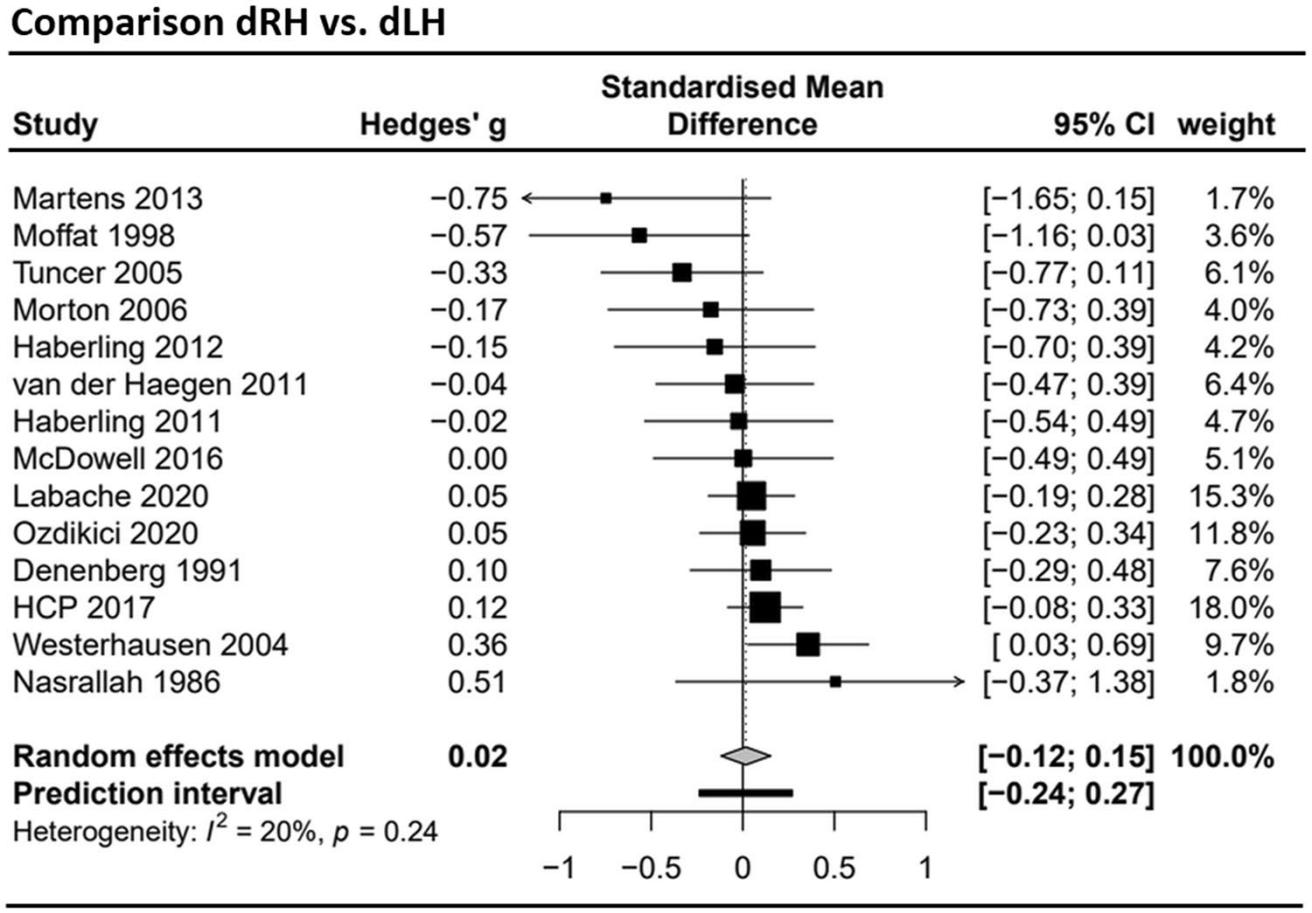
Forest plot of the meta-analysis of studies comparing dominant right hand (dRH) and dominant left hand (dLH) samples (dependent variable: total corpus callosum size). The total sample size across all *k* = 14 studies was *n* = 1910 for dRH and *n* = 646 for dLH sample. Negative values indicate the dLH group to have a larger corpus callosum, positive values indicate the dRH group to have a larger corpus callosum. HCP 2017 = Human Connectome Project, data release 2017 (see also Van Essen et al. 2013)

Concerning the total corpus callosum area, effect sizes from *k* = 14 studies were included in the meta-analysis, which incorporated data from 1910 dRH and 646 dLH participants. Details on the included studies and data extraction can be found the Supplement Table S1. As shown in the forest plot (Fig. 3), the estimated mean effect size *g* = 0.016 did not deviate significantly from zero (*t* = 0.27, *p* = 0.79). The 95% confidence interval (CI 95%) for *g* ranged from − 0.12 to 0.15. The between-study variance was *τ*^2^ = 0.01, suggesting a 95% prediction interval from − 0.24 to 0.27 around the mean effect. Neither inspection of the funnel plots (see Supplement Fig. S2) nor the Egger’s test of the intercept (*a* = − 1.35, *t* = − 2.01, *p* = 0.07) suggested a small study bias.

Meta-analyses of the subsectional data were not conducted as the number of studies available was below the minimum determined by the a priori power analysis. An overview of the available studies and their effect sizes can be found in Supplement Fig. S4.

### Meta-analysis set for the cRH vs. NcRH comparison

**Fig. 4:**
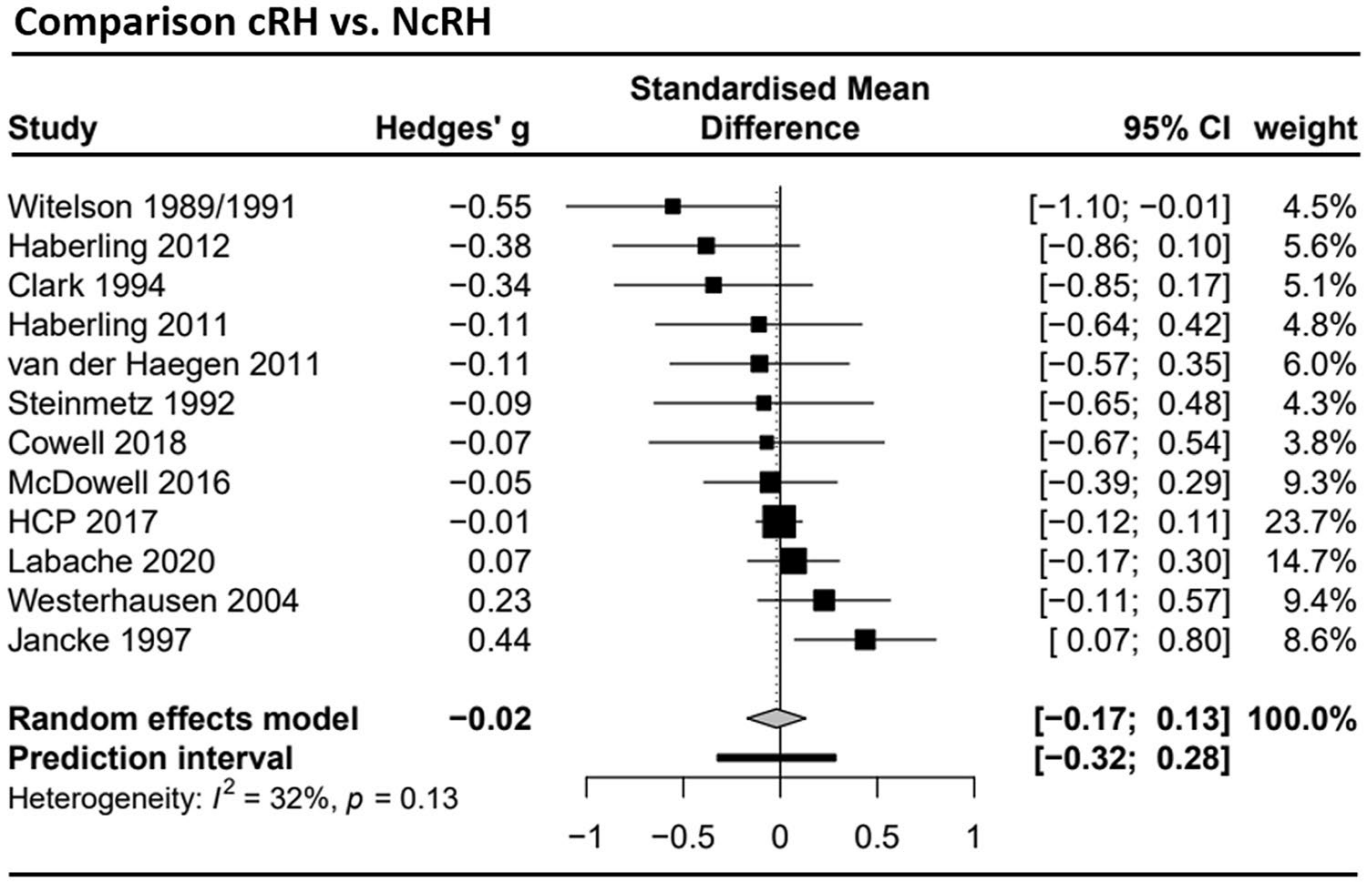
Forest plot of the meta-analysis of studies comparing consistent right-handers (cRH) and non-cRH (NcRH) (dependent variable: total corpus callosum size). The analysis included *k* = 12 studies with a total sample of *n* = 1149 for dRH and *n* = 1121 for NcRH. Negative values indicate the NcRH group to have a larger corpus callosum, positive values indicate the cRH group to have a larger corpus callosum. HCP 2017 = Human Connectome Project, data release 2017 (see also Van Essen et al. 2013)

Data from *k* = 12 studies (see Supplement Table S2) with a total of 1149 cRH and 1121 NcRH participants were included in the meta-analysis regarding the total corpus callosum area. The estimated mean effect size *g* = − 0.02 (CI 95% − 0.17; 0.13) did not deviate significantly from zero (*t* = − 0.28, *p* = 0.78; see forest plot Fig. 4). The heterogeneity between studies was *τ*^2^ = 0.01, resulting in a prediction interval from − 0.32 to 0.28. Inspection of the funnel plots (see Supplement Fig. S2) and the Egger’s test (*a* = − 0.63, *t* = − 0.95, *p* = 0.37) did not indicate a small study bias.

**Fig. 5:**
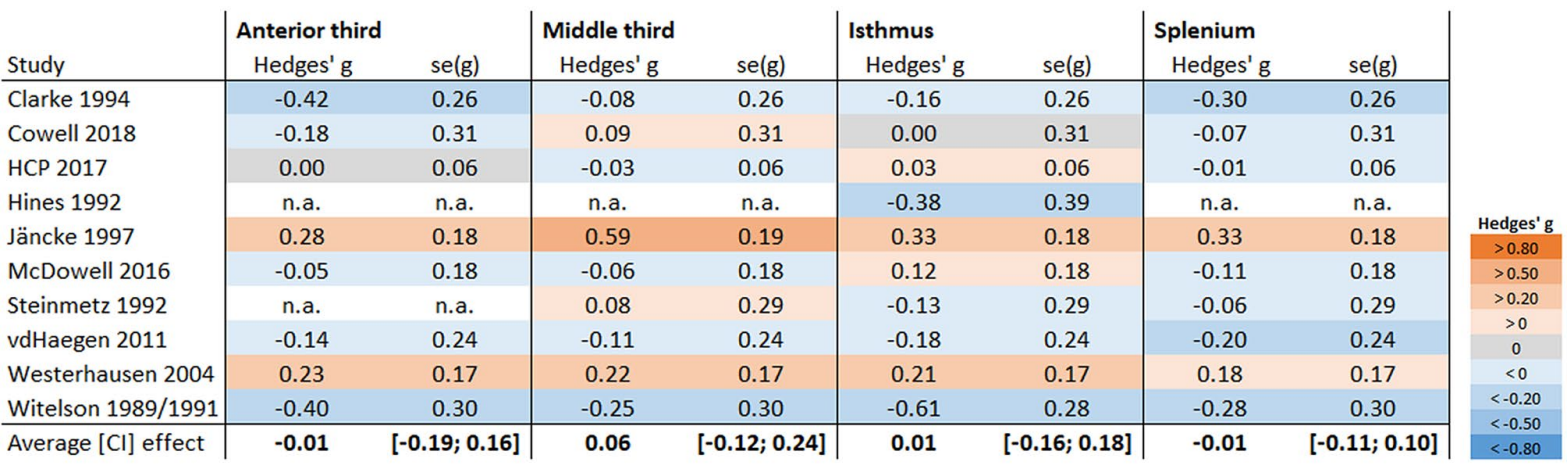
Overview of subsection meta-analyses comparing cRH and NcRH samples. The graph presents the effect size (Hedges’ *g*) and standard error of the effect size (se(*g*)) for each study. Negative values indicate the subsection to be larger in the NcRH group, positive values indicate the subsection to be larger in the cRH group. The provided average effect is estimated using a random-effects model. The values in brackets are the 95% confidence interval. Color coding was based on the Cohen’s effect-size heuristics (Cohen 1992) as indicated in the figure legend. Note, for some studies data was not available (n.a.) for some of the subsections. HCP 2017 = Human Connectome Project, data release 2017 (see also Van Essen et al. 2013)

Meta-analyses for all four subsections were conducted, but none suggested a significant group effect (see Fig. 5). Concerning the anterior third subregion, the meta-analysis included *k* = 8 studies (cRH: *n* = 946; NcRH: *n* = 848) and estimated a mean effect of *g* = − 0.01 (CI 95% − 0.19 to 0.16; *t* = − 0.19, *p* = 0.85; *τ*^2^ = 0.01; prediction interval: − 0.31 to 0.28). For the middle third, the analysis included *k* = 9 studies (cRH: *n* = 965; NcRH: *n* = 881) and estimated a mean effect of *g* = 0.06 (CI 95% − 0.12 to 0.24; *t* = 0.77, *p* = 0.46; *τ*^2^ = 0.02; prediction interval: − 0.32 to 0.44). Regarding the isthmus, *k* = 10 studies (cRH: *n* = 985; NcRH: *n* = 896) yielded a mean effect of *g* = 0.01 (CI 95% − 0.16 to 0.18; *t* = 0.12, *p* = 0.91; *τ*^2^ = 0.01; prediction interval: − 0.29 to 0.31). Finally, the mean effect for the splenium region (*k* = 9; cRH: *n* = 965; NcRH: *n* = 881) was *g* = − 0.01 (CI 95% − 0.11 to 0.10; *t* = − 0.14, *p* = 0.89; *τ*^2^ < 0.01; prediction interval: − 0.12 to 0.10). All four subsection meta analyses were repeated excluding the HCP data, as this sample contributed with a weight larger 25%. However, the estimated mean estimates were comparable to those above suggesting that the findings are not dominated by the HCP sample (see Supplementary Material Sect. 10 for details).

The additionally conducted subgroup analyses for the variable Sex did neither for the total corpus callosum (*Q* = 0.09; *df* = 1; *p* = 0.77) nor for the isthmus subsection find a significant difference (*Q* < 0.01; *df* = 1; *p* = 0.94). Regarding the total corpus callosum, the analysis included *k* = 10 datasets of a female subgroup, which was characterized by a *g* = 0.06 (CI 95% − 0.05 to 0.16; *τ*^2^ < 0.001). The male subgroup analysis contained *k* = 9 datasets, yielding a *g* = 0.03 (CI 95% − 0.19 to 0.25; *τ*^2^ = 0.02). Regarding the isthmus, the female subgroup analysis included *k* = 10 datasets and suggested a mean effect size of *g* = 0.04 (CI 95% − 0.11 to 0.18; *τ*^2^ < 0.01). The male subgroup analysis (*k* = 8 datasets) found a *g* = 0.03 (CI 95% − 0.25 to 0.31; *τ*^2^ = 0.03). The forest plots of both analyses are presented in Supplement Fig. S3.

### Meta-analysis set for the cRH vs. cLH comparison

**Fig. 6:**
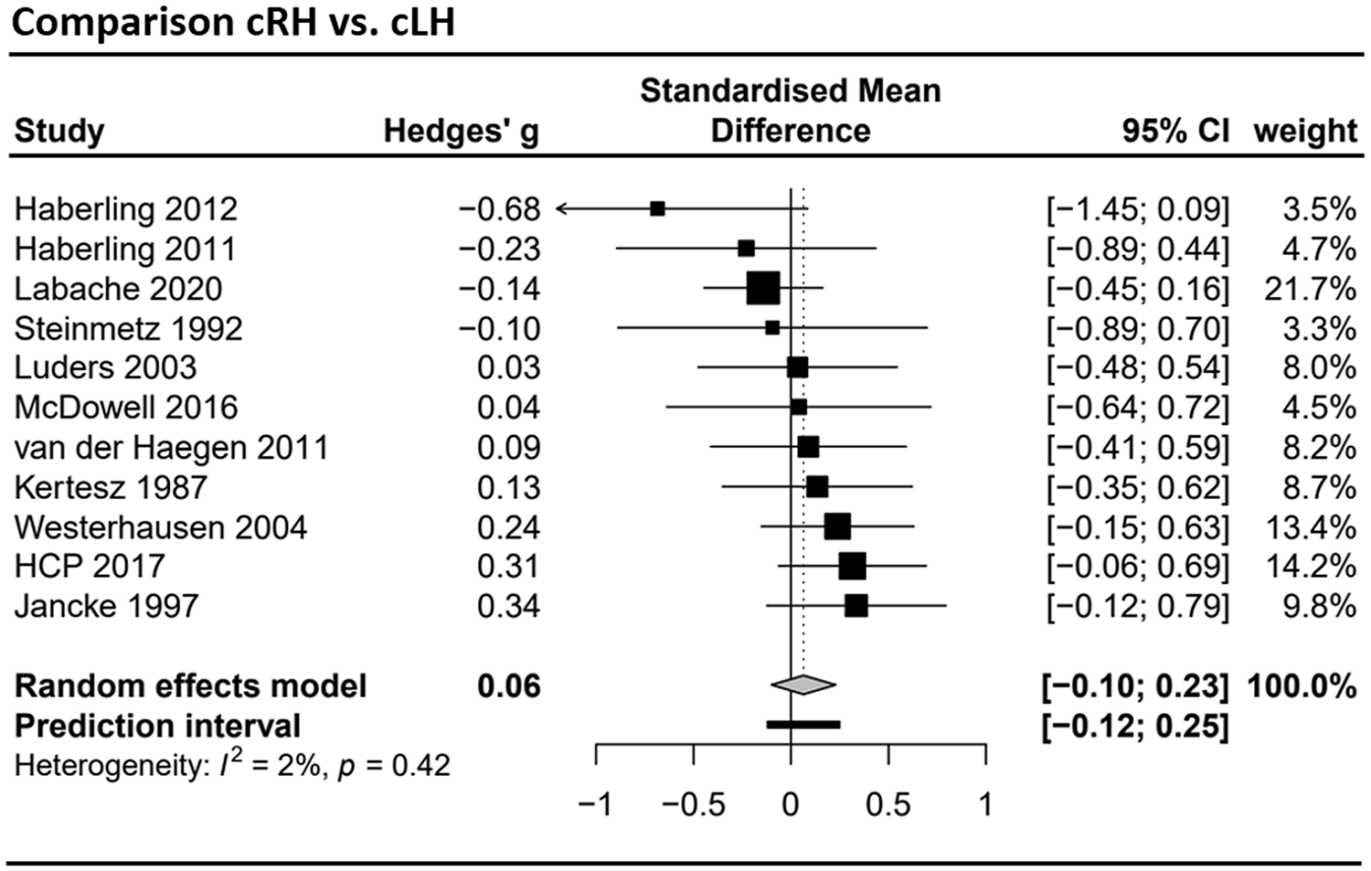
Forest plot of the meta-analysis of studies comparing consistent right-handers (cRH) and consistent left-handers (cLH) (dependent variable: total corpus callosum size). It included *k* = 11 studies with a total sample of *n* = 1142 cRH and *n* = 306 cLH participants. Negative values indicate the cLH group to have a larger corpus callosum, positive values indicate the cRH group to have a larger corpus callosum. HCP 2017 = Human Connectome Project, data release 2017 (see also Van Essen et al. 2013)

The data of *k* = 11 studies (see Supplement Table S3) was included in the cRH vs. cLH meta-analysis summarizing the data from a total of 1142 cRH and 306 cLH participants. The group difference in total corpus callosum size was estimated to be *g* = 0.06 (CI 95% − 0.10; 0.23) and did not deviate significantly from zero (*t* = 0.87, *p* = 0.41). As can be seen in the forest plot (Fig. 6), the heterogeneity between studies was with *τ*^2^ < 0.01 comparatively low, leading to a narrow prediction interval of − 0.12 to 0.25. Neither funnel plots nor Egger’s regression (*a* = − 1.08, *t* = − 1.01, *p* = 0.34) suggested a small study bias. Subsectional meta-analyses were not conducted as the number of studies available was below the minimum determined by the power analysis (see Supplement Fig. S4 for an overview).

### Meta-analysis set for the cRH vs. MH comparison

**Fig. 7:**
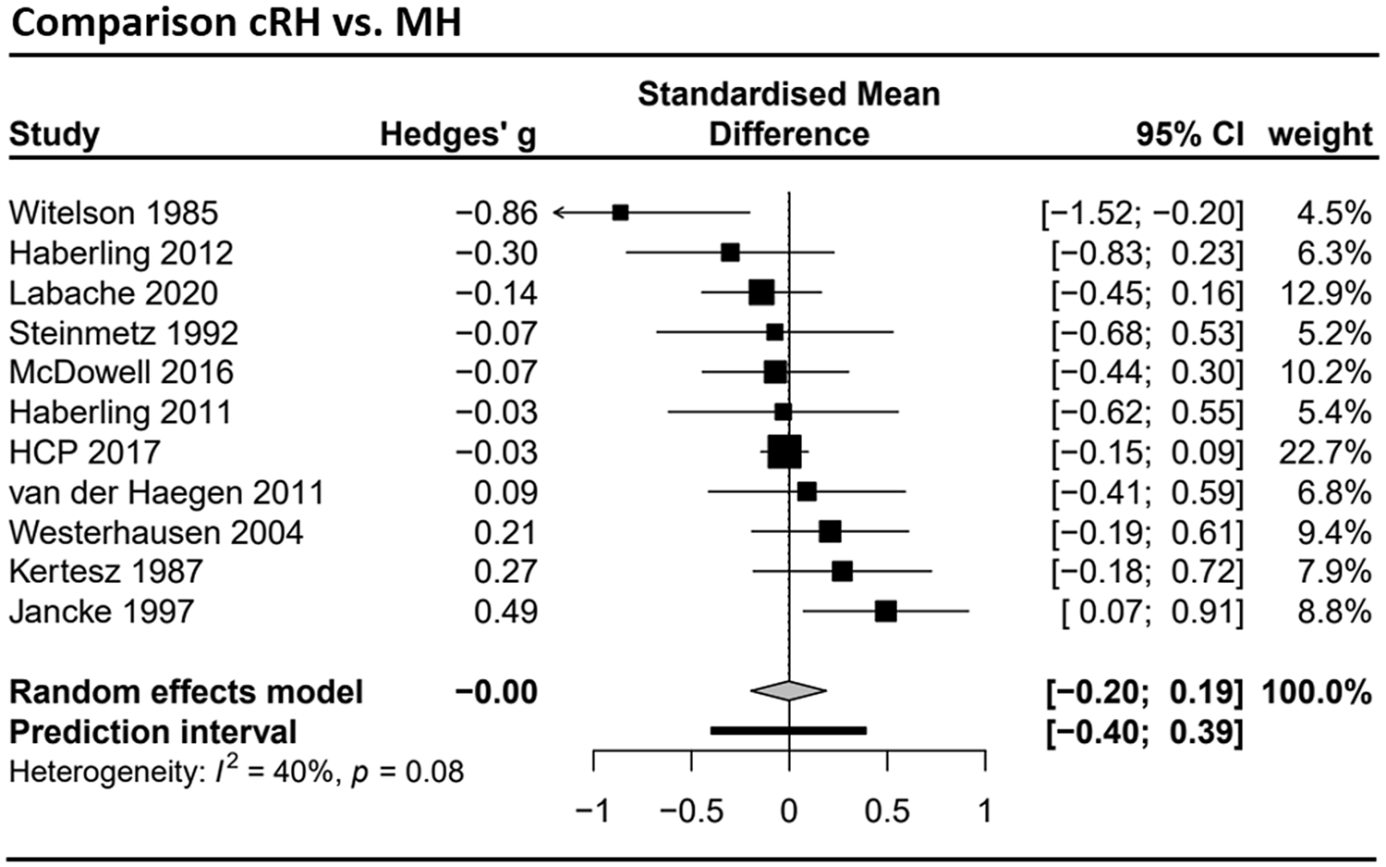
Forest plot of the meta-analysis of studies comparing consistent right-handers (cRH) and mixed-handers (MH) (dependent variable: total corpus callosum size). The analysis included *k* = 11 studies with a total sample of *n* = 1139 cRH and *n* = 810 MH participants. Negative values indicate the MH group to have a larger corpus callosum, positive values indicate the cRH group to have a larger corpus callosum. HCP 2017 = Human Connectome Project, data release 2017 (see also Van Essen et al. 2013)

The meta-analysis contrasting cRH and MH groups integrated data from *k* = 11 studies (see Supplement Table S4 and forest plot in Fig. 7) with data from a total of 1139 cRH and 810 MH participants. The differences in total corpus callosum size between the groups was estimated to *g* = − 0.004 (CI 95% − 0.20; 0.19) and was not statistically significant (*t* = − 0.05, *p* = 0.96). Between study variance was estimated to *τ*^*2*^ = 0.02 so that the 95% prediction interval was − 0.40 to 0.40. No indication for a small study bias was found (Egger’s regression: *a* = − 0.06, *t* = − 0.08, *p* = 0.94; see funnel plot in Supplement Fig. S2). A subsection analysis was only conducted for the splenium subsection (*k* = 7; cRH: *n* = 909; MH: *n* = 661). The mean effect size for the splenium analysis was *g* = 0.0005 (CI 95% − 0.28 to 0.28; see also Supplement Fig. S4) and was not significant (*t* = 0.00, *p* = 0.996; *τ*^2^ = 0.03; prediction interval: − 0.55 to 0.56).

## Discussion

The present study investigated the presence of differences in corpus-callosum morphology between individuals of different hand preference, using meta-analytic techniques. Irrespective of the nature of the compared hand-preference groups, we did not find a significant association of handedness and corpus-callosum morphology, neither for total corpus callosum size, nor for any of its subsections.

The interpretation of these non-significant findings requires first to consider the sensitivity of the conducted analyses. This can be done by referring to the confidence limits of the mean estimates, which provided a range of population effect sizes which cannot be reliably excluded by the available data. Considering the meta-analyses dealing with total corpus callosum size, the largest effect size included in the confidence limits was *g* = 0.23 as the upper boundary of the cRH-cLH comparison, which translates to an *r*^2^ = 0.009. Thus, this meta-analysis can exclude population effects which are larger than roughly 1% of the explained variance. The same applies to the other comparisons, as the confidence limits include even less extreme values. The meta-analyses of the sub-sectional data were comparatively less powerful resulting in wide confidence limits (see e.g., Fig. 5) as a result of the smaller number of studies and reduced overall sample size. Of note, however, also for the isthmus region, which had shown the largest difference between cRH and NcRH in Witelson’s original studies, effects larger |*g*|> 0.18 (*r*^2^ = 0.008) appear unlikely.

Thus, taken together, the present meta-analyses of differences in total corpus callosum size can be taken to exclude the existence of population effects larger than 1% explained variance with reasonable confidence. These effects would be considered “small” following Cohen’s effect-size conventions (Cohen 1992). Thus, the population effect sizes that cannot be excluded may be seen as being irrelevant and handedness-related differences in corpus callosum size could be considered negligible for cognition and behavior. On the other hand, as pointed out by Smith (2005), comparatively low amount of explained variances may have a substantial behavioral significance if they accumulate over many events. This may be the case with regard to the corpus callosum, considering its central role in the integration and coordination of information processing between the cerebral hemispheres. Thus, while the present analyses support the exclusion of comparatively small population effect sizes, we are hesitant to attribute functional insignificance to the effect size as it cannot be excluded. Any such conclusion would demand a better understanding of the relationship between variations in size and callosal functioning than currently available. Nevertheless, population-effect sizes of medium to large sizes, as were suggested by Witelson’s original work, can be excluded by the present analyses. Thus, we were not able to confirm the original findings neither in terms of the size of the effect nor in terms of statistical significance.

A closer inspection of the forest plots (Figs. 4 and 7) shows that the effect sizes of Witelson’s findings fall outside the 95% confidence interval of the estimated effect size for the respective meta-analysis as well as the prediction interval. The reasons explaining this strong deviation from the effects reported by other studies might be found in several characteristics of Witelson’s studies (Witelson 1985, 1989; Witelson and Goldsmith 1991). That is, the data was collected from autopsy specimen (rather than from in-vivo MRI), the study sample was comparatively old, and it was consisting only of (non-CNS) cancer patients. While each of these variables might affect callosal measurements, it appears, however, unlikely that these factors introduce systematic differences between right- and non-right handers. For example, while aging certainly has an effect on corpus callosum morphology (Danielsen et al. 2020; Doraiswamy et al. 1991; Hasan et al. 2008; Salat et al. 1997; Skumlien et al. 2018), the magnitude of aging-related callosal atrophy reduction would have to be more pronounced in NcRH individuals to produce the effect reported by Witelson. Likewise, cancer treatment (e.g., chemotherapy) negatively affects brain white-matter integrity, including the corpus callosum (Deprez et al. 2012), but to the best of our knowledge there is no evidence to suggest that NcRH are more resilient to these adverse side effects. An alternative explanation might be found when looking at the mean corpus callosum area reported across the three original studies. While the study sample was step-wise expanded by Witelson, the total corpus callosum area reported in particular for the male NcRH subsample decreased from a (comparatively high) value of 800.6 mm^2^ (sd: 53.9; *n* = 5) in 1985, to 786.3 mm^2^ (sd: 111.6; *n* = 6) in 1989, and to 744.0 mm^2^ (sd: 109.0; *n* = 9) in 1991. At the same time, all other subsample means stayed relatively stable. Therefore, one might speculate that outliers in the initial NcRH sample might have driven Witelson’s findings. Thus, Witelson’s finding of a larger corpus callosum in NcRH/MH might have been the consequence of a sampling bias.

Another issue that deserves attention is that the present null findings contrast a previous meta-analysis by Driesen and Raz (1995), which found a small effect size of Cohen’s *d* = − 0.13 (CI 95% − 0.23 to − 0.02), suggesting a significantly larger corpus callosum area in non-right handers. This meta-analysis included data from seven studies (Clarke and Zaidel 1994; Kertesz et al. 1987; Nasrallah et al. 1986; O’Kusky et al. 1988; Steinmetz et al. 1992; Witelson 1989; Yoshii and Duara 1989), of which the present analyses included five (excluded are the following two studies: (O’Kusky et al. 1988), data only presented for patients with epilepsy, and Yoshii and Duara (1989), published in Japanese). A closer look at the Driesen and Raz (1995) meta-analysis, however, also reveals some differences in the chosen approach. For example, the authors did not account for differences in handedness classification, and ignored, for example, that Nasrallah et al. (1986) compares dRH and dLH participants, while others (Kertesz et al. 1987; Steinmetz et al. 1992; Witelson 1989) compare cRH with NcRH samples. While this lack of specificity is certainly the consequence of the sparsity of studies available in 1995, which prevented a more sophisticated distinction, some inconsistencies in the data extraction remain difficult to explain. For example, in Table 3 (p. 243) Driesen and Raz list an effect size of Cohen’s *d* = 0.10 for the Steinmetz et al. (1992) study which would indicate larger areas in right-handers. Looking at the data provided in the original study, it rather is the case that the right-handers had the smallest corpus callosum compared to both MH and cLH groups. Furthermore, Driesen and Raz (1995) report an effect size of *d* = − 0.57 for a study by O’Kusky et al. (1988). O’Kusky et al. (1988) do not report relevant group differences for the corpus callosum between left- and right-handers. The study only provides a correlation of *r* = − 0.13 of handedness LI and callosal area for the studied sample of epileptic patients which might be converted to *d* = − 0.26. Consequently, it is not immediately clear where the effect size used in the Driesen and Raz meta-analysis originated.

One limitation of the present meta-analyses is that we were not able to evaluate handedness-related callosal differences controlling for brain-size differences, as the original studies typically did not consider brain size in their analyses or did not report relevant means or test statistics required for a meta-analysis. Thus, it can only be speculated how accounting for brain size might affect the handedness comparison. On the one hand, corpus callosum size and brain size are correlated positively (Jancke et al. 1997) so that possible brain-size differences between handedness groups might potentially act as a confounding variable. On the other hand, neither total brain volume nor white or gray matter volume have been found to differ between handedness groups in previous studies (Good et al. 2001; Hervé et al. 2006; Jancke et al. 1997; Luders et al. 2010; Witelson 1989), making a systematic effect less likely. Arguably, most informative for this question are studies reporting analyses of both raw and brain-size corrected data allowing for a direct comparison. Four of these studies did not find any obvious differences for the handedness effect on total corpus callosum size when the correction is applied (Jancke et al. 1997; Mitchell et al. 2003; Nasrallah et al. 1986; Witelson 1989). For example, Witelson (1985) reports a significant larger callosal area in NcRH than cRH both before and after including brain weight into her analysis. Only Hopper et al. (1994) claim in a table legend (i.e., without providing more details) that the body of the corpus callosum was found to be significantly larger in cRH compared to NcRH only after correction for brain size. However, summarizing the above, it appears unlikely that correcting for brain size would substantial change the conclusions compared to the analysis of the raw callosal measures. Nevertheless, it remains for future, large-scale studies to confirm statistically that the effect that hand preferences have on callosal morphology is neither moderated nor confounded by brain size.

A second variable that needs to be considered is the participants’ sex, as it may act as both moderator and confounding variable. Sex may be considered a moderator variable, as a series of early studies report an interaction of sex and handedness when predicting total callosal and, in particular, isthmus size (Burke and Yeo 1994; Clarke and Zaidel 1994; Denenberg et al. 1991; Habib et al. 1991; Witelson 1989). That is, a significantly larger isthmus area in NcRH compared to cRH has been found in male but not female participants (Witelson 1989, or  Habib et al. 1991, using data corrected for brain size). Burke and Yeo (1994) utilizing the raw *LQ* score, report a positive correlation of the hand preference and posterior callosal area measures in their male subsample (suggesting that more consistent handedness is associated with a larger structure), while a negative association was reported in the female subsample. However, the present meta-analyses do neither for total nor isthmus area support the notion of an interaction. First, across both meta-analyses neither males nor females showed a significant handedness effect for the cRH-NcRH comparison. Second, the direct comparison of the effect sizes did not yield a significant difference between males and females. Interpreting these findings, one has to keep in mind that the test power of the sex-specific and moderator analyses is reduced compared to omnibus analyses as fewer studies and smaller samples were included. Moreover, the power of moderator variables analysis itself is typically low within meta-analyses. Thus, arguably, more datasets would be beneficial to improve the meta-analyses’ sensitivity when it comes to the overall effect estimate, but even more so when it comes to the moderator variables analysis. Nevertheless, the overall pattern of findings provided no evidence for a strong interaction of sex and handedness when explaining corpus callosum morphology.

Meta-analyses and large-scale studies usually find the corpus callosum to be comparable between the sexes or slightly smaller in males once differences in brain size have been accounted for (Driesen and Raz 1995; Eliot et al. 2021; Smith 2005). For the here relevant uncorrected raw measures, however, the male corpus callosum can be expected to be bigger (Bishop and Wahlsten 1997; Luders et al. 2014; Smith 2005). Thus, differences in the proportions of male and female participants in the compared handedness groups might potentially bias the comparison and act as confounding variable. However, a look at the available data (see Tables S1–S4), indicates that the proportions typically were matched well between the compared handedness groups. Nevertheless, to further explore the possibility of a confounding influence of sex, we conducted supplementary meta-regression analyses using the difference in the proportion of females between handedness groups as a covariate. As can be seen in the Supplementary Material Sect. 9, for neither the dRH-dLH nor the cRH-NcRH comparison did we find a significant moderation effect. Thus, while differences in the proportions of males and females might affect the heterogeneity in the data, the differences in proportions are small and do not produce obvious meta-regressive effects, so that we do not believe that these have significantly affected the estimation of the mean effects.

Witelson emphasizes in her studies the relevance of consistency rather than the direction of hand preference (Witelson and Goldsmith 1991). Best comparable with this approach is the meta-analysis comparing cRH and MH participants, which did not yield a significant effect. However, it also deserves to be mentioned that a small series of studies chose a slightly different approach to the topic by comparing a group of consistent hand preference—containing both cRH and cLH participants—with a non-consistent group (McDowellet al. 2016; Welcome et al. 2009) or which used absolute *LQ* values in a correlative approach (Habib et al. 1991; Luders et al. 2010). While the group comparisons did not yield any significant association, Habib et al. (1991) found a significant positive correlation, indicating that more consistent individuals had a larger total corpus callosum area (Spearmann *r*_sp_ = 0.297, *p* = 0.03; *n* = 53). Luders et al. (2010), correlating callosal thickness measures with |*LQ*|, found a cluster of negative associations in the middle third of the corpus callosum (*n* = 361, of which 37 were dLH), thus showing the opposite association to Habib et al. To further explore this association, we conducted a supplementary analysis of the samples of which raw data was available (Labache et al. 2020; McDowell et al. 2016; Van der Haegen et al. 2011; Westerhausen et al. 2004). As can been seen in Supplementary Material Sect. 10, the Spearman correlations of total corpus callosum area and |*LQ*| for these four samples ranged form *r*_sp_ = − 0.15 to 0.10, with none of the correlations being significant. Although we refrained from conducting a meta-analysis for these studies, as their number was small, the available evidence does not support the existence of a substantial association of hand preference consistency and callosal measures.

Another consideration is that the present analysis focuses on morphological measures of the corpus callosum and will not be sensitive to differences on the microstructural level. While combined morphological and histological analyses suggest that the midsagittal area is a good predictor of the number of myelinated axons in the corpus callosum (Hou and Pakkenberg 2012; Riise and Pakkenberg 2011), studies comparing the diffusion characteristics of the corpus callosum between handedness groups could provide additional information. Three previous studies indeed suggest stronger anisotropy in the corpus callosum of non-right handers compared to right-handers (McKay et al. 2017; Westerhausen et al. 2004, 2003); all comparing cRH and cLH), potentially suggesting a higher average axon or myelin density in the corpus callosum. Other studies, however, failed to find comparable differences (Haberling et al. 2011; Peled et al. 1998) revealing inconsistency in the literature. Unfortunately, the number of available studies is limited, so that we were not able to conduct a meta-analysis of diffusion data.

Handedness may be assessed via self-report preference questionnaires or via differences in left- and right-hand performance (skill) in manual tasks (Tapley and Bryden 1985). As both do not necessarily correlate highly with each other (Corey et al. 2001), we originally had the intention to perform a separate meta-analysis of studies utilizing measures of hand skill for the assessment of handedness. However, we were only able to identify three such studies so that we refrained from conducting a statistical integration. Kertesz et al. (1987) correlated performance differences between the right and the left hand in a tapping test with total corpus callosum size and did not find a significant association (*r* = 0.07, *n* = 104). Steinmetz et al. (1995) used a paper-and-pencil manual tracing test to classify individuals into dLH (*n* = 58) and dRH individuals (*n* = 62), and did not find a significant difference in corpus-callosum size corrected for forebrain volume. Finally, Preuss et al. (2002), using the same manual tracing test as Steinmetz et al (1995), classified a group of nominal right-handed individuals into cRH (*n* = 32) and NcRH sample (*n* = 14), and did not find a significant difference in total callosal size or in any subsection. Thus, while the available evidence does not indicate that corpus-callosum differences can be found when handedness is determined via hand performance measures, the number of studies is small and more evidence is required before a conclusion can be reached.

Finally, the following potential biases from selective reporting and excluded studies deserve consideration when interpreting the findings. Firstly, for most of our meta-analyses concerning the total corpus callosum, a small-study bias is not indicated (through funnel plots and Egger’s regression analysis). Nevertheless, some studies, especially when analyzing subsectional data, selectively reported findings based on theoretical considerations (i.e., excluded regions from the analysis as previous studies did not find differences in these regions; e.g., Steinmetz et al. (1992) and Witelson and Goldsmith (1991)) or statistical significance (Hines et al. 1992; Martens et al. 2013; Moffat et al. 1998). In the latter case, this results in underreporting of non-significant, presumably small effect sizes, biasing the meta-analyses to overestimate the population-effect sizes (even when not noticeable in funnel plots). Thus, the effect-size estimates as shown in Fig. 5 need to be interpreted with caution considering this bias. Second, data from several relevant publications had to be excluded as necessary information or data was missing (for details see Supplement Table S5) or not available even after contacting the authors. While the findings of several of these studies were included in the discussion, it has to be emphasized that most of these excluded studies indicate non-significant differences between handedness groups. Thus, it appears likely that the results of the present meta-analyses overestimate the size of the population-effect sizes. However, as none of the meta-analyses yielded significance in the first place, this overestimation of the effect size is unlikely to have affected the conclusions drawn from the present meta-analyses.

In summary, the general interpretation of Witelson’s findings (Witelson 1985, 1989; Witelson and Goldsmith 1991) that non-consistent handedness is associated with stronger inter-hemispheric connectivity, is not supported by the present meta-analyses of corpus callosum morphology. About 35 years after the first publication, the original findings have been rendered unreliable by the research they inspired. Thus, any assumption about callosal connections that is made based on hand preference are invalid. However, future large-scale studies are required to allow for a more powerful evaluation of sex differences, brain-size effects, as well as performance measures when studying handedness effects in the corpus callosum.

## Supplementary Information

Below is the link to the electronic supplementary material.

Supplementary file1 (PDF 1435 KB)

## Declarations

### Availability of data and material

The extracted mean values and standard deviations that served as the basis of the present meta-analyses are available in tabulated form on the accompanying OSF platform (https://osf.io/sw6ev/). The PRISMA checklist is also available on this platform.

### Code availability

The R scripts used for running the presented analyses are also available on the OSF platform (https://osf.io/sw6ev/), and allow for reproduction of all results.
